# Host Factors of Favorable Intestinal Microbial Colonization

**DOI:** 10.3389/fimmu.2020.584288

**Published:** 2020-10-07

**Authors:** Sabine Pirr, Dorothee Viemann

**Affiliations:** ^1^Department of Pediatric Pneumology, Allergology and Neonatology, Hannover Medical School, Hanover, Germany; ^2^PRIMAL Consortium, Hanover, Germany; ^3^Cluster of Excellence RESIST (EXC 2155), Hannover Medical School, Hanover, Germany

**Keywords:** neonatal mucosal immunity, developing microbiota, shaping of microbiota, gut microbial colonization, host factors

## Abstract

Gut microbial colonization starts with birth and initiates a complex process between the host and the microbiota. Successful co-development of both establishes a symbiotic mutual relationship and functional homeostasis, while alterations thereof predispose the individual life-long to inflammatory and metabolic diseases. Multiple data have been provided how colonizing microbes induce a reprogramming and maturation of immunity by providing crucial instructing information to the newborn immune system. Less is known about what host factors have influence on the interplay between intestinal immunity and the composition of the gut microbial ecology. Here we review existing evidence regarding host factors that contribute to a favorable development of the gut microbiome and thereby successful maturation of gut mucosal immunity.

## Introduction

**Figure 1:**
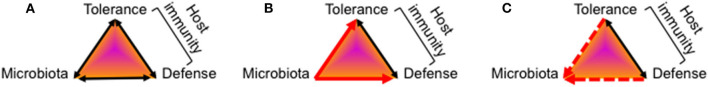
Mutual relationship between the host's immune system and the gut microbiota. The function of the host's gut mucosal immune system toward colonizing microbes is determined by the net sum of tolerance and defense activities which is depicted in a model of a triangular relationship. All elements of the triangle can be impacted by diverse environmental factors not included in this model. **(A)** Healthy gut colonization and immune adaptation mutually co-develops into a stable, energy-efficient state of homeostasis between the host's immune system and the microbiota. **(B)** Multiple evidence exists that after birth colonizing microbiota induce maturation of gut mucosal immunity by activation of tolerance-mediating signaling pathways as well as antimicrobial defense programs. **(C)** The knowledge on how the host's immune system at the beginning of life impacts on the kind of colonizing microbes and the further development of the microbiota composition is still fragmentary.

Several hundred bacterial species colonize the human gastrointestinal tract and live in a mutual relationship with the host and its immune system (1, 2). A healthy adult gut microbiota is stable and aids in digestion, detoxification and as a barrier against pathogens (3, 4). Importantly, the development of a health gut microbiota keeps the host immune system in a balanced state of tolerance and defense activity (Figure 1A). Though still ill defined, dysbiotic microbiome states are characterized by an altered microbiota composition of loss of diversity resulting in a bloom of pathobionts, loss of commensals, shift in metabolic capacities, and associate with the development of highly prevalent chronic inflammatory and metabolic diseases and/or an increased susceptibility to infections (5–9).

The postnatal period is particularly critical for the establishment of a healthy gut microbiota composition that promotes the postnatal maturation of gut mucosal and extra-intestinal immunity (10–15). The character of resulting host–microbe interactions determines what degree of gut homeostasis is achievable. Hornef and Torow recently proposed to implement the concept of the “neonatal window of opportunity” into the model of “layered immunity” as a timed succession of non-redundant phases during postnatal immune system maturation and establishment of host-microbial immune homeostasis (16). However, it often remains elusive whether a host factor or a microbiota state evolves as a result from the impact of the respective other feature or whether it represents a true driving force in this deeply interwoven developmental process. Clarification of this question is challenging and requires studies focusing on the beginning life with a high and longitudinal time resolution of effect sizes and directions.

Multiple data, especially those from experiments involving fecal transplants, demonstrated how colonizing microbes induce a reprogramming and maturation of immunity by providing crucial instructing information to the newborn immune system (10, 17–20) (Figure 1B). The importance of intestinal bacterial colonization for normal immune system development became particularly obvious by studies in germ-free mice that are lacking several immune functions (21–23) or the comparison of wild and laboratory mice (24). A surrogate in humans is antibiotic treatment that was shown to affect microbiome development of neonates and infants and translates into short- and long-term disorders characterized by dysregulated or impaired immune functions (25–33).

More challenging is the elucidation of the role of the host's state of immunity for the development of the gut microbiome (Figure 1C), especially that of infant-derived immune factors compared to maternally provided immune factors that supplement or impact on the infant's immune system. Moreover, the mode of action is often not clearly detangled in terms of microbiota-shaping by direct antimicrobial activity or indirectly via priming or regulation of neonatal mucosal immunity which then in turn impacts on the colonization or expansion of specific microbial communities. Finally, many studies have identified factors with immunoregulatory functions for the newborn's immune system but without further demonstration of a link to the microbiome development (those works will therefore not be discussed here).

Of course, exogenous factors such as the mode of delivery, infant feeding patterns, maternal diet, and environmental factors including drugs are also well-known to impact on the microbiome development either directly or indirectly by modulating the host's state of immunity. While these important microbiota-shaping exogenous directors have been extensively discussed elsewhere (34–36), we will focus in this review on endogenous host factors. Maternal (Table 1) and infant-derived (Table 2) host factors will be highlighted and evaluated regarding the present level of evidence on their role in shaping the development of the gut microbiome in humans and animals and thereby the maturation of gut mucosal immunity.

**Table 1:** Studies providing evidence for the influence of maternal host factors on the postnatal development of the infant's gut microbiome.

**Host factor^a^**	**Study subjects**	**Microbiota-shaping effect derived from association studies**	**Microbiota-shaping effect experimentally or per RCT^b^ validated**	**Mode of microbiota-shaping action^c^**	**References**
**Breast milk**					
IgA	human, mouse	yes	yes	direct and indirect	(37–40)
HMOs^d^	human, mouse, hamster, pig	yes	yes	direct (*indirect?*)	(35, 41–50)
α-Lactalbumin	human, mouse	inconclusive	inconclusive	direct	(51–54)
Lactoferrin	human, pig	inconclusive	inconclusive	direct	(55–57)
Lysozyme	mouse, pig	yes	yes	direct	(58–60)
TGF-β	human	yes	no	unknown	(61)
S100A8/A9	human, mouse	yes	yes	direct and indirect	(62, 63)
Extracellular vesicles	mouse	no	yes	indirect	(58, 64)
Human milk microbiome	human, mouse	yes	no	direct (*indirect?*)	(65–69)
**Obesity**	human	yes	no	unknown	(70, 71)

a *Factors supplied by the mother and produced endogenously in the neonatal intestine are highlighted*.

b *RCT, randomized controlled clinical trial*.

c *Direct by antimicrobial or prebiotic activity or indirect by priming of mucosal immunity or unknown*.

d *HMO, human milk oligosaccharides*.

**Table 2:** Studies providing evidence for the influence of infant-derived host factors on the postnatal development of the infant's gut microbiome.

**Host factor^a^**	**Study subjects**	**Microbiota-shaping effect derived from association studies**	**Microbiota-shaping effect experimentally or per RCT^b^ validated**	**Mode of microbiota-shaping action^c^**	**References**
**Host genotype**	human, mouse	yes	no	unknown	(72–76)
**Innate immune signaling**					
TLR5	mouse	yes	yes	indirect	(77, 78)
IRAK-1	mouse	inconclusive	inconclusive	indirect	(79)
TLR4 (S100A8/A9)	human, mouse	yes	yes	direct and indirect	(62, 63)
NOD2	human, mouse	yes	no	unknown	(75, 80–82)
NLRP3	mouse	yes	inconclusive	unknown	(83)
NLRP6	mouse	yes	yes	*indirect?*	(84–88)
**Antimicrobial peptides**					
Defensins	mouse	yes	yes	direct	(89)
Reg3^d^	mouse	inconclusive	yes	direct	(90–94)
Mucins	mouse	yes	yes	direct	(95–98)
**Antimicrobial proteins**					
Lactoferrin	human, pig	inconclusive	inconclusive	direct	(55–57)
Lysozyme	mouse, pig	yes	yes	direct	(58–60)
**Others**					
IgA	human	yes	no	direct and indirect	(40, 99–101)
TGF-β	human	yes	no	unknown	(61)
miRNAs	mouse	yes	yes	direct (*indirect?*)	(102–104)

a *Factors supplied by the mother and produced endogenously in the neonatal intestine are highlighted*.

b *RCT, randomized controlled clinical trial*.

c *Direct by antimicrobial or prebiotic activity or indirect by priming of mucosal immunity or unknown*.

d *Reg3, regenerating islet-derived protein 3 family*.

## Maternal Factors

### Breast Milk

Human breast milk is the ideal nourishment for infants and has been shaped by evolution to contain essential nutrients and immunological components necessary for the neonate during its initial phase of life. Numerous studies indicate that breastfed neonates are better protected against infections, inflammatory disorders and allergies and show improved cognitive development compared to infants fed with formula (105). In the following we report human breast milk components of different substance classes with the strongest evidence for a capacity to modulate the developing microbiota either directly via antimicrobial or prebiotic activities or indirectly by modulating immune functions of the host.

#### Immunoglobulin A (IgA)

During the first months of life, breast milk is the predominant source of IgA as B cells generally do not populate the intestine until about 4 weeks of age (37, 106). In mice, early exposure to maternal soluble IgA via breast milk prevented the translocation of aerobic bacteria from the neonatal gut into draining lymph nodes (38). In human preterm infants, a high ratio of IgA-coated *Enterobacteriaceae* to uncoated *Enterobacteriaceae* was associated with a lower risk of NEC while a mouse model confirmed the NEC-protective effect of breast milk IgA (37). It is speculated that secretory IgA contributes to the creation of temporal and spatial niches and exerts a strong selection pressure on colonization along the gastrointestinal tract. IgA levels are particularly high in crypts. Agglutination and immune exclusion might primarily act against highly prevalent bacteria and thus favor colonization by minorities and microbial diversity (107). In addition, secretory IgA binds to components of the mucus (108) and co-localizes specifically with gut bacteria in the outer mucus layer (109). The outer mucus layer forms a distinct microbial niche colonized by bacteria with mucolytic capability (110). IgA-coated bacteria might be retained in the mucus layer, which reduces their shedding and promotes diverse colonization (107). In neonatal mice, secretory IgA dampened T cell-dependent immune responses against commensal bacteria (39) and was involved in the selective suppression of Proteobacteria during establishment of the early gut flora (40). A reduced IgA supply via breast milk during infancy came along with an altered gene expression profile of intestinal epithelial cells characterized by an increased expression of genes associated with intestinal inflammatory diseases in humans (38). By the age of weaning, the induction of a Gammaproteobacteria-specific IgA response partially contributes to the transition from a neonatal to a mature microbiota (40). Experiments in germfree mice showed that colonization with microbes from mice lacking IgA have persistent increased colonization with Gammaproteobacteria that results in sustained intestinal inflammation and increased susceptibility to neonatal and adult models of intestinal injury (40). Collectively, there is clear evidence in mice and humans that that IgA supplied via breast milk has direct and indirect beneficial effects on the developing gut microbiota.

#### Human Milk Oligosaccharides (HMOs)

HMOs are the third largest solid component of breast milk after lipids and lactose and known to have a bifidogenic effect on the infant's microbiome. HMOs promoted the growth of specific Bifidobacteria, supporting an early Bifidobacteria-dominated gut microbiome (35, 41, 42). The structure of some HMOs resembles that of epithelial pathogen receptors, enabling them to serve as a decoy receptor to prevent pathogen binding and enhance pathogen clearance (42). A mother's secretor status is genetically defined and determines the HMO concentration and profile in her breast milk (111–113). The abundance of Bifidobacteria in the intestinal microbiome of breastfed infants of non-secretor women was lower than in that of children of secretor women (43, 44). In preterm infants, colonization with *Bifidobacterium breve* was shown to depend on the composition of HMOs but not the mother's secretor status (45). Infant fecal HMO concentrations change over time and correlate with a shift in the fecal microbiota from a non-saccharolytic population dominated by commensals of the birth canal to a population dominated by saccharolytic microbes (46) Several randomized controlled trials showed that infants fed with a formula supplemented with the HMOs 2′-FL ± lacto-N-neotetraose or galacto-oligosaccharides develop a distinctive stool bacterial population compared to infants fed a control formula which is more similar to that of breastfed infants (increased colonization with Bifidobacteria and *Lactobacillus* and decreased colonization with pathobionts) and was accompanied by a lower plasma inflammatory cytokine concentrations (47–50). *In vitro*, HMOs prevented the intestinal epithelial attachment of enteropathogenic *Escherichia (E.) coli* but whether this was a direct or indirect effect remained elusive (114) With respect to the mode of action, HMOs were shown to modulate also toll-like receptor (TLR)-dependent proinflammatory signaling, while elevating cytokines involved in tissue repair and homeostasis (115–117). However, these effects could not be linked to the gut microbiota-shaping effect so far. Further mechanistic studies are required to understand how HMOs influence the development of the gut microbiota.

#### α-Lactalbumin

α-Lactalbumin is the major whey protein in breast milk and is digested in the small intestine (118). Several peptides released from α-lactalbumin during digestion have been proposed to elicit biological effects. *In vitro* studies demonstrated their antimicrobial activity toward potential pathogens as *E. coli, Klebsiella pneumoniae, Staphylococcus aureus, Staphylococcus epidermis, Streptococci*, and *Candida albicans* as well as their prebiotic activity by stimulating the growth of Bifidobacteria (51–53). A study in mice showed significantly higher proportions of *Lactobacillus, Parabacteroides* and *Bifidobacterium* in animals fed a high-fat diet supplemented with α-lactalbumin compared to a control diet (54). However, only a few data have been published on the effects of α-Lactalbumin on developing gut microbiota communities in humans and remain inconclusive (119, 120).

#### Lactoferrin

Lactoferrin is a non-heme iron binding protein with direct microbial as well as immunomodulatory activities. Lactoferrin is digested to some extend in the infant's intestine yielding bioactive peptides (121). Degradation products of lactoferrin such as lactoferricin (122–125) or Lf(1–11) and lactoferrampin (126, 127) also showed strong bactericidal activity against various kinds of both Gram-positive and Gram-negative bacteria and might contribute to the following net effect of lactoferrin. Piglets fed recombinant human lactoferrin had a greater abundance of *Lactobacillus* and lower abundances of *Veillonella* and *Escherichia-Shigella* in the jejunum and of *Actinobacillus* in the ileum at 1 week resp. 3 weeks of life (55). Several *in vitro* studies have shown that human lactoferrin is able to stimulate the growth of Bifidobacteria (56, 57). *In vivo*, a bifidogenic effect of lactoferrin has also been suggested, but only for bovine and not for human lactoferrin (128), or based on a non-confounder-controlled association between fecal lactoferrin levels on day 3 of life with the amount of fecal Bifidobacteria and Lactobacilli in human infants (129). Clinical trials on the microbiota-shaping effect of the nutritional supplementation of newborn infants with bovine lactoferrin have reported conflicting results with no significant impact on the developing fecal microflora (130, 131) or reduction of pathobionts in a very small cohort of 21 very low birth weight preterm infants (132) or a minor contribution to the total Bifidobacterium population in 202 healthy term infants (133). Human lactoferrin exerts direct antibacterial and antiviral activities through iron depletion (134), interaction with microbial and target host cell surfaces (135) and by preventing the entry of viral particles into the host cells, either by blocking cellular receptors or by directly binding to the viral particles (136). Furthermore, lactoferrin modulates innate and adaptive immune responses thereby controlling the expression of different anti- and pro-inflammatory cytokines and chemokines [e.g., interleukin (IL)-4, IL-10, IL-1, IL-6, IL-12, tumor necrosis factor-α] which impacts on the growth, differentiation, activation and function of immune cells (136–138). Whether and how the immunomodulatory effects contribute to the microbiota-shaping effect or whether the latter primarily results from the antimicrobial activity of lactoferrin remains to be shown.

#### Lysozyme

Lysozyme is an antimicrobial protein secreted by glandular epithelial cells that functions by cleaving bacterial peptidoglycan, causing a loss of cellular membrane integrity and cell lysis. In adult sows, feeding of high amounts of lysozyme reduced the intestinal richness of *E. coli* and increased abundance of *Lactobacillus amylovorus* (58). Recombinant human lysozyme expressed at high level in milk of transgenic pigs inhibited the growth of *E. coli* in the duodenum of piglets (59). In transgenic mice, expression of recombinant human lysozyme promoted the growth of *Bifidobacterium* and inhibited the growth of *Salmonella* in the intestine (60). In humans, the role of lysozyme for a developing gut microbiome is unknown.

#### Transforming Growth Factor β (TGF-β)

There is only one study showing that breast milk cytokines TGF-β1 and TGF-β2 partially explains variation in gut microbial composition among human breast-fed neonates. Especially, high concentrations of TGF-β2 were associated with a richer microbiome with an increased abundance of several bacteria, including members of Streptococcaceae and Ruminococcaceae, and lower relative abundance of distinct Staphylococcaceae taxa in neonatal gut composition (61). The exact mode of the microbiota-shaping effect has not been clarified and experimentally validated yet.

#### S100A8/A9

The alarmins S100A8 and S100A9 are myeloid-related proteins. Upon extracellular release they typically form the heterodimer complex S100A8/A9, also known as calprotectin (139). Human breast milk contains exceeding high amounts of S100A8/A9 with the highest levels found after the vaginal delivery of a full-term neonate compared to birth of a preterm infant per cesarean section (62). S100A8/A9 proved to be an important mediator of the antimicrobial activity of breast milk, especially inhibiting the growth of manganese sensitive bacteria such as *Staphylococcus aureus* and group B streptococci (62). Willers et al. found that human infants with reduced levels of fecal S100A8/A9 during the first year of life had an altered development of the gut microbiome characterized by less abundant Actinobacteria including Bifidobacteriaceae but dominant colonization with Gammaproteobacteria, particularly opportunistic Enterobacteriaceae (63). This gut microbial state in turn translated into a higher risk of neonatal sepsis and development of obesity (33, 63). It could furthermore be demonstrated in mice that the high enteral supply of S100A8/A9 after birth primes a regulatory phenotype in lamina propria macrophages with a high expression of Cx3cr1, Il-10, and Tgf-β, that is followed by a stronger expansion of regulatory T cells in intestinal tissues and a restricted expansion of Enterobacteriaceae. A single feeding of S100A8 after birth prevented the excess expansion of Enterobacteriaceae as well as fatal sepsis in neonatal mice (63).

#### Extracellular Vesicles

Extracellular vesicles in breast milk may influence the establishment of the intestinal microbiota by altering the local immune response toward challenges from microbial colonization (105). Breast milk exosomes contain proteins, RNA, microRNA and long non-coding RNA (140–142). Profiling studies found these microRNAs to be related to multiple biological functions of a wide range of immunological pathways (143, 144). Likewise, proteomic analyses revealed that human breast milk extracellular vesicles include proteins involved in regulation of cell growth and controlling inflammatory signaling pathways (142). In addition to vesicles released by maternal cells (exosomes), a recent study revealed the presence of bacteria-derived extracellular vesicles in breast milk (145). Their vertical transfer or indirect involvement into the development of the infant's gut microbiota is possible but has not been shown yet. The presence of serum-derived extracellular vesicles impacted on epithelial and immune cell responses to the gut microbes *Lactobacillus* or *Bifidobacterium* and enhanced their aggregation and phagocytosis and modulated TLR-induced cellular responses (64). Whether breast milk extracellular vesicles have similar effects is not known yet. Feeding of bovine milk exosomes to young adult mice altered bacterial communities in the murine cecum (higher abundance of Tenericutes and Lachnospiraceae, lower abundance of Verrucomicrobiaceae) (58). Data on the influence of milk extracellular vesicles and their cargo on human infant's gut microbial communities are still lacking.

#### Human Milk Microbiota

Although not in a strict sense an endogenous maternal factor, the mother the human milk microbiota is at least mother-specific and represents therefore for the infant an endogenous factor in the broader sense. Human milk contains ~10^6^ bacterial cells/mL. Commonly isolated microbiota in human milk are *Staphylococcus, Streptococcus, Propionibacterium, Bifidobacterium*, and *Lactobacillus* among others (146–150). The composition and variation of the human milk microbiota are associated with the maternal body mass index, parity, glucose tolerance status, mode of delivery, ethnicity, breast feeding practices, and other milk components (151, 152). The origin of bacteria in human milk is not well-established. Two different routes, which are probably not mutually exclusive, have been proposed: (i) surface skin contamination and retrograde flow during breastfeeding and (ii) translocation through a more speculative gut-mammary route (153). The assumption that microbes in breast milk contribute to the establishment of the infant intestinal microbiome is supported by several studies in mother-infant pairs using sequencing techniques and demonstrating that breast milk and term infants' feces share specific microbial strains of *Bifidobacterium, Lactobacillus, Enterococcus*, and *Staphylococcus* (65–69). *In vitro* and mouse studies demonstrated that several *Lactobacillus* strains isolated from human milk exert immune-modulatory effects on bone marrow-derived macrophages as well as antibacterial properties against pathogenic bacteria (149, 150). However, clear evidence for a colonization of human milk microbiota in the infant's intestine or an indirect impact of human milk microbiota on the developing intestinal microbiome has not been provided yet. Kim and Yi recently raised the question whether extracellular vesicles derived from the human milk microbiota are involved in the vertical transfer from mothers to their progeny (145), which remains unanswered to date.

### Obesity

Neonates born to mothers with obesity showed a 50% reduction in the intestinal abundance of Gammaproteobacteria at 2 weeks of age compared with infants of normal-weight mothers (70). Furthermore, infants born to overweight mothers were found to have an increased abundance of *Bacteroides* and *Staphylococcus* at 1 and 6 months of age compared to infants of normal-weight mothers (71). Reasons might be the different and less diverse bacterial community in breast milk from mothers with obesity compared to normal-weight mothers, including higher levels of *Staphylococcus* and *Akkermansia muciniphila* and lower levels of *Bifidobacterium* (154, 155). Differences in the composition of hormones, cytokines and HMOs might also play a microbiota-shaping role (156, 157). These findings might even challenge the beneficial value of breast milk feeding in case of overweight mothers. This concern is corroborated by the data that antibiotic use in infants born to mothers with obesity reduced the risk of obesity, whereas antibiotic use in infants born to normal-weight mothers increased the risk of obesity (158). Another reason for the altered microbiome composition in infants born to obese mothers might be iron deficiency (157, 159, 160). Mothers with obesity and those with excess gestational weight gain as well as their infants are significantly more often iron deficient (161, 162). Several clinical and *in vitro* studies have demonstrated the impact of iron deficiency and supplementation on the gut microbiota composition and function and intestinal inflammation in infants and children (163–168). However, whether iron deficiency is the causative mechanism altering the postnatal gut microbiota colonization in infants of mothers with obesity needs to be further explored.

## Infant Factors

### Host Genotype

There is growing scientific evidence indicating that host genetics influence the acquisition and development of the infant gut microbiota (72–74). Studies in mice revealed that host genetics in the interplay with the diet shape the intestinal microbiota composition with individual genes and regulatory circuits only beginning to be identified (72, 73). Benson et al. found 18 quantitative trait loci in mice that show significant or suggestive genome-wide linkage with the relative abundances of specific microbial taxa (72). A study using three large human cohorts established a clear association between the host genotype and the gut microbiota state in adulthood. In this analysis, 58 single nucleotide polymorphisms (SNPs) at nine loci were associated with microbial taxa and a total of 33 loci were associated with functional units, with 21 loci associated with metabolic pathways and 12 loci associated with gene ontogeny categories. For example, variants of the LCT locus responsible for the human lactase production linked to the abundance of Bifidobacteria, while variants of the NOD2 locus had an impact on the pathway abundance of enterobactin biosynthesis which in turn strongly correlated with the abundance of *E. coli* (75). Another study in a large human twin cohort uncovered associations between heritable taxa and genes related to diet, metabolism, olfaction, barrier defense, and self/non-self-recognition (76). Summarized, linkage studies on associations between genetic variants and gut microbiota states point to possible important host factors that are crucial for the establishment of a healthy gut microbiome. For most of presently identified genetic trait loci further research is needed to confirm the relevance of these host factors for the development of the gut microbiome experimentally and in human infants.

### Innate Immune Signaling

#### Toll-Like Receptors (TLRs)

Age-dependent differences exist for the intestinal expression of individual TLR receptors in mice and humans (77, 169). This fact already suggests TLR signaling playing a role for the postnatal development of the gut microbiome and evidence for this specific relationship is accumulating. Several mouse models with knock-downs or genetic variants targeting TLR-signaling pathways at different levels (*MyD88, Tlr2, Tlr4, Tlr5, Tlr9, Trif* , NF-kB-pathway) are characterized by spontaneous or permissive colitis (20, 170–181). We learned from these mouse models how critical commensal-mediated TLR-signaling is for the training and maintenance of gut mucosal immunity and integrity. The host's primary phenotype and programming of TLR-signaling is conversely probably likewise important for the development of the gut microbiome. However, this reverse effect order has been clarified only for a few TLR-related host factors and barely in humans.

For the flagellin receptor **TLR5** it has been clearly shown that its transient expression on the gut epithelium of neonatal mice contributes to the establishment of a favorable gut microbiota that lowers the risk of intestinal inflammatory injuries (77, 78). Fulde et al. demonstrated in mice that a TLR5-mediated epithelial production of the regenerating islet-derived protein 3 γ (REG3γ) is critical to restrict the colonizing of flagellated bacteria. This host-mediated regulatory circuit of bacterial colonization acts solely during the early neonatal period but influences life-long microbiota composition. The relevance of TLR5-signaling on the gut microbiota development in human infants has not been demonstrated yet.

Chassin et al. demonstrated that the microRNA-146a-mediated translational repression and proteolytic degradation of the essential TLR signaling molecule interleukin 1 receptor associated kinase 1 (**IRAK1**), an integral element of MyD88-dependent TLR-signaling pathways, induces intestinal epithelial innate immune tolerance toward lipopolysaccharide (LPS) of Gram-negative bacteria. It furthermore protects murine neonates against epithelial damage from oral challenge with *E. coli* (79), suggesting that IRAK1 has a beneficial impact on the established microbiota. In contrast, however, 16S rRNA profiling of stool samples revealed miR-146a-deficient mice having a rather favorable gut microbiota composition protecting against *Listeria monocytogenes* infection compared to wild-type mice with decreased levels of the Proteobacteria phylum, *Prevotellaceae* family, and *Parasutterella* genus, and significantly increased short-chain fatty acid producing bacteria, including the genera *Alistipes, Blautia, Coprococcus_1*, and *Ruminococcus_1* (102). Thus, the role of IRAK1 for the development of the gut microbiota remains to be clarified and also elucidate in humans.

The alarmins **S100A8** and **S100A9** are endogenous **TLR4** ligands (182). Next to their high supply via breast milk (62), S100A8 and S100A9 are also endogenously produced in intestinal tissues by lamina propria macrophages, in infants at significantly higher levels than in intestinal tissues from adults (63). Studies in *S100a9* knock-out mice and cross-fostering settings delineated the mode of action. S100A8/A9-mediated TLR4-signaling during the neonatal period tolerized the inflammatory responsivity of lamina propria macrophages and increased the expression tonus of regulatory genes like IL-10 and transforming growth factor-β. This regulatory macrophage phenotype impacted on the developing microbiota in mouse and humans as outlined above. To achieve full effects regarding immunoregulation and modulation of gut colonization the additional supply of S100A8/A9 via breast milk was mandatory (63).

#### NOD-Like Receptors (NLRs)

Similar as in the case of TLRs, murine NLR knock-out models (*Nod2, Nlrp3, Nlrp6, Nlrc4*, caspase 1) with increased (albeit partly controversial) susceptibility in different colitis models (183–188) point to NLRs as important host factors for the establishment of a protective gut microbiota. However, for most factors this assumption awaits experimental approval.

Mutations in the peptidoglycan sensor **NOD2** locus represent one of the most important genetic risk factors for inflammatory bowel disease (IBD) (189, 190). As mentioned above, genetic variants of the NOD2 locus in humans correlated with the intestinal abundance of *E. coli* (75). In two cohorts of adult patients with IBD, a significant positive correlation was found between the number of IBD risk alleles in the NOD2 exon and the relative abundance of *Enterobacteriaceae* (80). Two recent studies in mice demonstrated that NOD2 influences microbial resilience with prolonged alterations of the microbiota in *Nod2*-deficient mice after antibiotic treatment, in case of neonatally treated mice resulting in an increased susceptibility to colitis in adulthood (81, 82). However, experimental evidence for NOD2 shaping the primary development of the gut microbiota in neonates and infants and the related mode of action is still lacking.

For **NLRP3** it could recently been shown that the compositions of the gut microbiota in *Nlrp3* knock-out mice and wild-type mice were significantly different (83). At the genus level, NLRP3 deficiency decreased the relative abundance of *Bacteroides* but increased the abundance of *Desulfovibrio, Mucispirillum, Oscillospira*, and *Ruminococcus*. Surprisingly, fecal microbiota transplantation from *Nlrp3* knock-out mice significantly ameliorated depressive-like behaviors of recipient wild-type mice in a chronic stress model suggesting that restriction of NLRP3-dependent inflammasome signaling is important to ensure healthy gut microbiota development. For a final evaluation of the role and mode of action of NLRP3 regarding its impact on developing gut microbiota compositions further clarifying data in mice as well as humans is desirable.

Elinav et al. were the first group demonstrating that the lack of intestinal epithelial **NLRP6**-dependent inflammasome activity results in an expansion of the bacterial phyla Bacteroidetes and TM7 and that this microbiota state exacerbates chemical colitis (84). This microbiota-shaping effect was later ascribed, but not directly linked, to a regulating impact of NLRP6 on mucus secretion by goblet cells (85). However, littermate-controlled mouse studies then dismissed the impact of NLRP6-dependent inflammasomes on the composition of the commensal gut microbiota (86, 87). This conflict could be detangled by showing that NLRP6 matters in dependence of the microbial community structure. In pathobiont-free conditions WT and *Nlrp6*-deficient mice had a similar microbiota while upon introduction of pathobionts a high prevalence, specifically of Prevotellaceae, Verrucomicrobiaceae, and Helicobacteraceae, in *Nlrp6*-deficient mice became obvious (88). This finding was also largely in line with the previous findings (84). Demonstration of the biological relevance of NLRP6 shaping the microbiota development in humans would now be convincing.

### Antimicrobial Peptides (AMPs)

The anatomical distribution of AMPs in mice and humans shows age-dependent differences (77, 191). In fetal intestine AMPs are already expressed prenatally and levels increase with gestational age (192, 193). Major AMP families are cathelicidins, α-defensins, and β-defensins, lectins, and mucins (194, 195). The peptides are stored in vacuoles of granulocytes and/or epithelial cells ready to be secreted upon microbial stimulation. In addition to their ability to kill bacteria by disrupting critical membrane functions, many AMPs have chemotactic and immunomodulatory activities (194, 196). It is evident that induced expression of AMPs is frequently an integral part of a response to a microbial challenge, reflecting how colonizing microbes train the host's immunity (197, 198). Conversely, though not in humans but at least in mice, there is also evidence that the levels and composition of AMPs in the gut can regulate the development of the microbiota.

In human **α-defensin 5** (HD-5) transgenic mice significant changes in the microbiota composition were observed compared to wild-type littermate controls, specifically a lower relative abundance of Firmicutes and higher percentages of Bacteroidetes (89). Human REG3α and its murine homolog REG3γ are C-type soluble, carbohydrate-binding lectins found throughout the small intestinal epithelium, and in the large intestine in response to infection and inflammation (199). Bacterial binding (90, 91) and membrane permeabilization (92) are proposed to be important for the bactericidal activity of REG3. Hitherto shown effects of REG3α resp. REG3γ on the gut microbiota are not consistent, most likely due to diverse confounders like REG3 expression levels, age, and initial health condition. In a murine model of alcoholic steatohepatitis, intestinal REG3γ was protective by reducing the mucosa-associated microbiota and preventing bacterial translocation (93). On the other side, the fecal microbiota of mice that express human REG3α in hepatocytes (hREG3α travels via the bile to the intestinal lumen) showed a significant shift in microbiota composition compared to control mice, with an enrichment of Clostrididales (Ruminococcaceae, Lachnospiraceae) and depletion of Bacteroidetes (Prevotellaceae) (94). These mice were less sensitive to colitis, suggesting that increased intestinal levels of REG3α might impair the development of a healthy gut microbiota state.

**Mucins** are the main structural components of mucus, the biopolymer found throughout the gastrointestinal tract (199). Some commensal intestinal bacteria, including Bacteroidetes, are mucolytic and use mucin glycoproteins as an energy source (95, 96). Therefore, mucins have been proposed to play an important role in shaping microbial communities at the intestinal luminal surface. Recent studies in mice suggest a correlation between changes in mucin glycosylation profiles and deviations of overall microbial community ecology as well as altered abundances of specific microbes (97, 98). Mice lacking terminal fucose residues in their distal gut mucins showed significantly higher abundance of members of the genera *Parabacteroides, Eubacterium, Parasutterella, Bacteroides*, and family *Lachnospiraceae* and lower abundance of an unclassified genus within the order Clostridiales (97). Changes in mucus glycosylation due to loss of core 1-derived O-glycans in mice led to inverse shifts in the abundance of the phyla Bacteroidetes and Firmicutes (98).

Summarized, further supporting studies in mice and particularly first evidences in humans are warranted to verify the host-microbiome direction of the AMP-microbiota axis.

### Antimicrobial Proteins

Antimicrobial proteins play important roles in the first line of defense, by either killing pathogens directly, or by contributing to an efficient immune response to eliminate pathogens. Most abundant antimicrobial proteins in humans are **lactoferrin** and **lysozyme**, both of which are also contained in breast milk. Their potential to influence the postnatal development of the gut microbiome composition has been discussed above. Whether the endogenous production of these proteins is important for the putative microbiota-shaping effect or to what extent the endogenous production contributes to the effect of the maternal supply of these factors has not been clarified so far. However, the lack of considering also the endogenous production and levels of these factors might have hampered the significance of effect sizes of the exogenous supply in previous studies.

### Others

#### Immunoglobulin A (IgA)

The knowledge on how IgA impacts on the developing gut microbiota is mainly based on studies with a focus on the IgA supply via breast milk and has been outlined above. However, also the endogenous IgA production by the infant is of relevance in this context. Patients with common variable immunodeficiency (CVID) showed a large shift in the microbiota (reduced abundance of Actinobacteria and Firmicutes and increased abundance of Gammaproteobacteria) which was particularly pronounced in patients with undetectable serum IgA and also found in patients with selective IgA deficiency (99–101). In patients with selective IgA deficiency, metagenomic analyses revealed pathobiont expansion (e.g., Gammaproteobacteria including *E. coli*) and depletion in some typically beneficial symbionts belonging to the Bacteroidetes and Firmicutes phylum (100, 101, 200, 201).

#### Transforming Growth Factor β (TGF-β)

TGF-β is a regulatory cytokine ubiquitously produced by immune cells and epithelial cells in intestinal tissues. While breast milk levels of TGF-β correlated with the neonate's microbiota composition (61), the role endogenously produced TGF-β plays in this context has not been explored so far.

#### MicroRNA (miRNA)

MicroRNAs are single-stranded non-coding RNA molecules with a length varying between 18 and 23 nucleotides that operate in post-transcriptional regulation of gene expression (202). miRNAs are found in various body fluids as plasma, urine and breast milk as well as in human feces (103, 143, 203, 204). Fecal miRNAs are secreted by intestinal epithelial cells (IEC), particularly homeodomain-only protein homeobox (HOPX)-positive cells, such as goblet and Paneth cells (103). *In vitro* studies revealed that specific fecal miRNAs were able to enter *Fusobacterium (F.) nucleatum* and *E. coli*, regulate bacterial gene transcripts, and affect bacterial growth. Furthermore, IEC-miRNA deficient mice showed uncontrolled gut microbiota compared to wild-type mice and exacerbated colitis. Wild-type fecal miRNA transplantation restored fecal microbes and ameliorated colitis (103). Moreover, the abundances of the phyla Bacteroidetes, Firmicutes, Actinobacteria, Bacteroidetes, Cyanobacteria, and Proteobacteria correlated with distinct murine miRNA levels (104). Another murine model revealed differing gut microbiota compositions with decreased levels of the Proteobacteria phylum, *Prevotellaceae* family, and *Parasutterella* genus, and significantly increased short-chain fatty acid producing bacteria, including the genera *Alistipes, Blautia, Coprococcus_1*, and *Ruminococcus_1* in miR-146a-deficient mice relative to wild-type mice (102). Whether this is an indirect effect linked to the miR-146a-mediated induction of intestinal epithelial innate immune tolerance (79, 205) or due to a direct microbial activity of miR-146a has not been detangled yet. Ji et al. demonstrated that human fecal miRNAs associated with inflammatory bowel disease have diverse effects on the proliferative activity of the intestinal bacteria *F. nucleatum, E. coli* and segmental filamentous bacteria following the uptake of these miRNAs by the microorganisms (206). Of note, data on the influence of fecal miRNAs on the developing intestinal microbiome in neonates and infants are missing to date. Moreover, for miRNAs found in breast milk (143) neither a microbiota-shaping effect nor correspondences with microbiota-shaping fecal miRNAs could be demonstrated so far (wherefore they have not been listed in Table 1 but highlighted in Table 2 as factors supplied by the mother and produced endogenously in the neonatal intestine).

## Concluding Remarks

Acknowledging that the establishment of a diverse and stable gut microbiota during the first years of life is crucial for life-long immune homeostasis, acquisition of tolerance, resistance against pathogens, and overall health fueled recent research work for factors that regulate the development of a health-promoting host-microbiota relationship. Present studies proposing host factors possibly important in this process of gut microbiome development differ regarding the therefore provided level of evidence. The spectrum of proof ranges from sole assumptions based on known biological functions, e.g., factors with antimicrobial activity, through association studies of host factor levels or genetic variants with specific microbiota states up to the experimental confirmation of the microbiota-shaping function, at best including delineation of the related mode of action. Moreover, the studies that provide functional data are mostly restricted to investigations in the mouse but lack demonstration of the biological relevance of the respective host factor in humans. Only if the latter condition is met together with a high grade of functional evidence for a beneficial microbiota-shaping effect this would allow and encourage translation of the concept into a clinical trial. Currently, only a few factors (IgA, HMOs, and S100A8/A9) meet these criteria. Further studies are needed to advance existing data or to unravel novel host factors important for a favorable postnatal development of the gut microbiome. Unveiling such endogenous mechanisms of microbiota regulation is important as they are highly attractive candidates for exploiting nature's principles for effective intervention strategies to ensure the development of health.

## Author Contributions

SP and DV equally conceptualized and composed the manuscript. All authors contributed to the article and approved the submitted version.

## Conflict of Interest

The authors declare that the research was conducted in the absence of any commercial or financial relationships that could be construed as a potential conflict of interest.
